# Molecular Modeling of Surfactant Interaction on Phospholipid Bilayers Mimicking Corneal Epithelium

**DOI:** 10.1021/acs.jctc.6c00863

**Published:** 2026-06-29

**Authors:** Isteaque Ahmed, Stephen J. Fox, C. Mark Maupin, Oreste Todini, Dilnoza Amirkulova

**Affiliations:** † 2514 Procter and Gamble Digital Accelerator at University of Cincinnati, Cincinnati, Ohio 45221, United States; ‡ Procter and Gamble, Reading Innovation Centre, Reading RG2 0RX, U.K.; § 6865 Pacific Northwest National Laboratory, Richland, Washington 99352, United States; ∥ Procter and Gamble, Brussels Innovation Center, 1583 Brussels, Belgium; ⊥ Procter and Gamble, Mason Business Center, Mason, Ohio 45040, United States

## Abstract

Surfactants found in consumer products can compromise eye corneal membrane integrity upon accidental exposure. Traditional in vitro and in vivo approaches to evaluate membrane–surfactant interaction pose experimental limitations such as species variability, reproducibility, and most often do not provide the overall picture. These limitations motivate the use of *in silico* models to study phenomena like cellular disruption assays caused by surfactants at the molecular scale. In this work, coarse-grained molecular dynamics simulations have been employed to investigate how nonionic alcohol ethoxylate (AE) and anionic surfactant alcohol ethoxy sulfate (AES) interact with lipid bilayer liposomes that mimic corneal epithelial cell membranes. The spherical liposome is composed of 1,2-dihexadecanoyl-*sn*-glycero-3-phosphocholine (DPPC), 1,2-di­(9*Z*-octadecenoyl)-*sn*-glycero-3-phosphoethanolamine (DOPE), 1,2-di­(9*Z*-octadecenoyl)-*sn*-glycero-3-phospho-l-serine (DOPS), and cholesterol, resembling the composition of the corneal epithelial cells’ membrane bilayer. The simulation consisted of varying degrees of representative surfactant compositions and two initial types of surfactant configurations within or outside the liposome. Our results reveal that both surfactants induce outer leaflet bulging, agreeing with membrane solubilization models. The more highly ethoxylated surfactant, AE, caused more consistent inner leaflet disruption than AES, resulting in significantly more water permeation and membrane thinning. In addition, both surfactants increase the lateral diffusion of lipids within the membrane layers, with higher ethoxylated AE showing a stronger effect than AES. This study demonstrates how surfactant structure and localization influence bilayer membrane integrity, offering mechanistic insights into the irritation potential, thus guiding the rational design of effective surfactant-based formulations.

## Graphical abstract

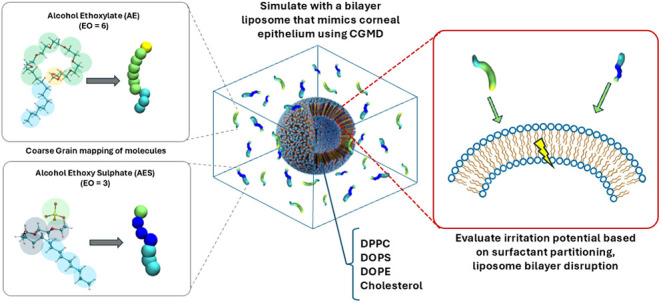

## Introduction

Understanding ocular responses to chemical irritants is crucial to the development of chemicals with reduced irritancy profiles and, ultimately, the formulation of milder personal care products.^1^ Just below the tear films, the corneal epithelial cells line the outer surface of the human eye. These are the first layer that come into contact with irritants.^2^ Historically, evaluation of the ocular response to chemical exposure required dependence on animal testing, such as the Draize test for rabbits, which raises ethical questions,^3,4^ or the isolated chicken eye (ICE) tests, which are slow and do not provide mechanistic insights. Consequently, in vitro and *in silico* models have emerged as powerful alternatives. Assays that specifically elucidate membrane-bilayer disruption are particularly ideal for studying these membrane-level interactions without the need for animal testing.

Particularly, epithelial cell membrane and liposome modeling play a pivotal role in fields that involve physiological process studies,^5,6^ tissue engineering,^7,8^ toxicology,^9^ drug development,^10,11^ and fundamental molecular research.^12^ These models aim to recapitulate the intricate architecture of epithelial tissues, encompassing both cellular constituents and the underlying extracellular matrix.^6,13^ Along with alternative in vitro assays for eye irritation evaluation, the development of *in silico* systems that closely approximate in vivo conditions has significantly reduced reliance on animal models. Molecular dynamics (MD) simulations leveraging such models have enhanced our understanding of membrane function and stimulus response mechanisms.

Liposome bilayers represent a crucial class of self-assembled structures composed of amphiphilic phospholipids, forming closed spherical vesicles with an aqueous core.^14^ These liposomes exhibit structural homology closely resembling the natural fluid-mosaic model lipid bilayers of epithelial cells.^9,15^ The structural foundation of these membranes is primarily composed of amphiphilic phospholipids, which spontaneously self-assemble into bilayers due to their hydrophilic headgroups and hydrophobic acyl chains.^16,17^ The specific composition and stoichiometry of these lipids significantly dictate membrane properties such as fluidity, permeability, and curvature of the cells or vesicles that these liposome surrogates are trying to emulate.^18^ Several studies detail the specific lipid profiles required to accurately model these epithelial barriers. For example, Kenchegowda et al. demonstrate that the corneal epithelium is predominantly composed of phosphatidylcholine (PC), phosphatidylethanolamine (PE), and sphingolipids, with oleic and palmitic acids acting as the major esterified fatty acids.^19^ The versatility of liposome vesicles allows for tailoring of their properties to mimic the mechanical responses of epithelial cell membranes under both physiological and pathological conditions.^20,21^ Furthermore, the lipid composition can be adjusted to mimic membrane functionality in terms of selective ion permeability,^22^ maintenance of cellular homeostasis,^23^ protein sorting,^24^ and signaling ordered phase behavior.^25^ These characteristics render liposome vesicles an apt candidate for substituting epithelial cell membranes in in vivo studies.^26^ The isolation and characterization of membrane components necessitate preparation,^18^ disruption,^27^ and solubilization of biological membranes.^28^ Surfactants play a pivotal role in these processes due to their ability to perturb the lipid bilayer of epithelial cell membranes through mechanisms such as endocytosis,^29^ membrane solubilization in protein isolation, or phase separation. The interaction between membranes and permeants, particularly surfactants,^30^ has been extensively investigated using a variety of biophysical and biochemical techniques.^31,32^ These investigations reveal and explain the multiple modes of surfactant–membrane interactions. For instance, cationic surfactants have increased the ion permeabilization and membrane disruption through electrostatic interactions with negatively charged membrane components.^22^ Conversely, anionic surfactants typically function as protein-denaturing agents with strong interaction with trans membrane proteins.^32^

Common surfactants such as cetyltrimethylammonium bromide (CTAB), sodium dodecyl sulfate (SDS), and Triton X-100 (TX-100) have been reported to disrupt bilayer membranes.^33^ Several pathways and models have been proposed to explain the mechanisms of surfactant-induced membrane disruption. Helenius et al. introduced a three-stage model that describes the process of membrane solubilization, focusing on thermodynamic equilibrium.^34^ In the first stage, free surfactant monomers rapidly partition into the membrane bilayer from the solution, a process dependent on the surfactant-lipid partitioning coefficient. The partitioning into the outer bilayer is faster than the slower partitioning to the inner layer, which is influenced by the surfactant’s headgroup characteristics. The second stage occurs at higher surfactant concentrations, when the surfactants saturate the lipid membrane, leading to membrane disintegration. In the final stage, at sufficiently high surfactant concentrations, the bilayer is fully solubilized and converted into mixed surfactant-lipid micelles; when the surfactant is in excess, these mixed micelles can coexist with surfactant-rich (pure) micelles in the solution. Lichtenberg et al. proposed a similar three-stage disruption mechanism emphasizing the kinetics of the surfactant molecules.^35^ This model specifies the formation of thread-like mixed micelle structures mediated by surfactant concentrations.

Surfactant headgroups are inherently curvophilic, meaning they form spontaneous curvature upon aggregation.^36^ In contrast, lipids in membrane bilayers are curvophobic and exhibit a flat surface.^37,38^ The process of membrane disruption by surfactants typically begins with the partitioning of free surfactant monomers into the liposome, initiating perforation.^37^ The rate and extent of this process depend on several factors, including the structural characteristics of the surfactant, particularly its headgroup size and polarity.^39^ Surfactants with bulky polar headgroups face challenges in traversing the membrane, leading to distinct disruption or perforation outcomes based on their lateral and vertical translocation.^40^ Notably, these membrane-disruptive effects often occur at surfactant concentrations below their critical micelle concentration (CMC), emphasizing the significance of surfactant monomer-membrane interactions in this process.^41^

Complementing experimental approaches, molecular dynamics (MD) simulations provide molecular-level insight into the dynamics and energetics of surfactant–membrane interactions.^42,43^ Because all-atom simulations of lipid membranes (particularly at the length and time scales required for membrane remodeling) are computationally demanding,^4^ coarse-grained (CG) models are widely used to access larger systems and longer time scales while retaining the essential physics of lipid self-assembly and membrane deformation.^28^ Prior CG studies provide important proof-of-concept for this approach. For example, Wanapinun et al. used CG MD to examine how the cyclic peptide kalata B1 perturbs lipid bilayers, showing stable peptide adsorption at the membrane–water interface and peptide-number-dependent curvature induction.^44^ Similarly, Shobhna et al. employed CG simulations to characterize ethanol-driven structural alterations of liposomes and reported good agreement with experimental observations.^45^ While these studies focused on different perturbants and membrane setups (e.g., planar bilayers or simplified lipid compositions) and did not target anionic/nonionic surfactants in corneal-epithelium mimicking spherical liposomes, they demonstrate that CG simulations can capture key membrane responses such as curvature generation, destabilization, and disruption. Building on these established methods, here we model a spherical bilayer liposome with a lipid composition chosen to approximate key features of corneal epithelial cell membranes and systematically investigate surfactant-dependent membrane remodeling across seeding configurations and concentrations.

In this work, we modeled a spherical bilayer liposome using a phospholipid composition that closely mimics the corneal epithelial cell membrane composition. Our aim was to develop a generalized simulation system to perceive the relationship and behavior of amphiphilic exogenous molecules and their potential to cause eye-membrane disruption. We employed CG MD simulation techniques to create and effectively simulate a large-scale system, enabling us to study complex membrane–surfactant interactions over extended time scales. Our approach optimized the initial configuration setup, allowing us to focus primarily on liposome-exogenous surfactant molecule interactions rather than micelle formation via phospholipids. We investigated both anionic and nonionic amphiphilic molecules to discern their lipid interaction behavior in driving the molecular initiation event that causes ocular irritation, providing molecular-level insights into the surfactant–liposome interaction. The findings are expected to be invaluable not only for designing more effective and improved surfactant-containing products but also for further removing barriers to modeling and simulating molecular systems that mimic those of organic structures. This research contributes to the growing body of knowledge on surfactant–membrane interactions and provides a foundation for future studies aimed at developing milder and more effective consumer products.

## Computational Method

### Initial Configuration

Although the exact phospholipid composition of the corneal epithelium remains a subject of debate, Table 1 summarizes several *in vitro* attempts to mimic this cell membrane (Rintoul, 1984; Zelenka,[Bibr ref46] 1978; Kapoor,^47^). For this study, we developed coarse-grained (CG) systems based on the composition described by Rintoul et al., as it aligns with an internal Procter and Gamble vesicle formulation previously validated to accurately mimic the corneal epithelium. Specifically, the lipid mixture comprises 1,2-dihexadecanoyl-*sn*-glycero-3-phosphocholine (DPPC, 64% by weight), 1,2-di­(9*Z*-octadecenoyl)-*sn*-glycero-3-phosphoethanolamine (DOPE, 17%), 1,2-di­(9*Z*-octadecenoyl)-*sn*-glycero-3-phospho-l-serine (DOPS, 14%), and cholesterol (CH, 5%).^48^ Lipid parametrization was followed according to standard MARTINI 2.0 ^49^ definitions and building-block rules. Exogenous surfactants included alcohol ethoxylate sulfate (AES) (*C* = 12, *n*_EO_ = 3) and alcohol ethoxylate (AE) (*C* = 12, *n*_EO_ = 6) (Table 2). Surfactants were seeded in two primary configurations: (a) in the outer leaflet of the liposome (denoted as inside liposome, outer leaflet (IS), Figure 1a,b) randomly seeded outside of the liposome (denoted as outer seeding (OS), Figure 1b). A liposome system with no surfactants was considered as the control. Three surfactant concentrations were considered for each surfactant and seeding (IS, OS) scenario. The concentrations for the outer-seeded surfactants were calculated on the basis of the critical micelle concentration (CMC) of their respective counterparts. The surfactant weights for these systems were considered as the percentage of total lipid weights, consisting of 1.3%, 6.52%, and 13.03% for AES and 1.3%, 6.89%, and 13.79% for AE. This methodology builds upon previous works demonstrating phospholipid, both single and bilayer liposome formation kinetics using CG simulations,^50^ while offering a novel approach to studying surfactant–membrane interactions in a more biologically relevant context. We added the detailed molecule counts for each system with appropriate counterions and water molecules in the supporting document (Table S1).

**Figure 1:**
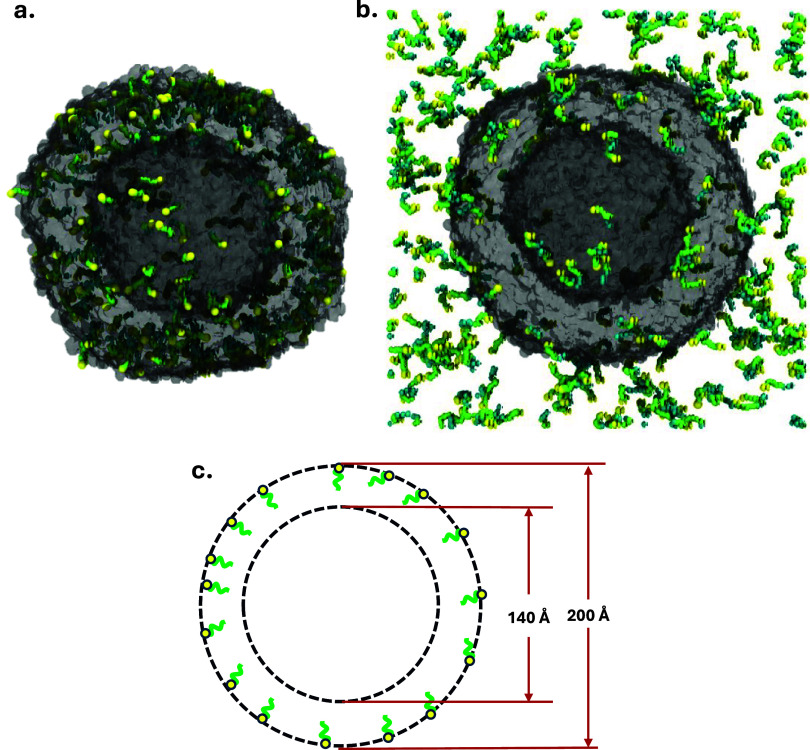
Schematic representations of minimized liposome structures—(a) Liposome with inside liposome-seeded (IS) surfactants, where the surfactants are seeded within the outer leaflet (Front view). (b) Liposome with outside liposome-seeded (OS) surfactants, where the surfactants are seeded within the bounding box and outside the liposome (Front view). (c) Inner and outer dimensions of the liposome.

**Table 1:** Phospholipid Bilayers Mimicking Corneal Epithelium Cell Membranes^a^

phospholipid	Zelenka et al.^46^ [wt %]	Kapoor et al.^47^	this work
PC	50	47	64
PE	15	35	17
PS	12	0	14
SM	12	0	0
Others (CH, PI, PG)	11	20	5

a Abbreviations: PC represents lipids with a phosphocholine headgroup, and PE: Phosphoethanolamine; PS: phospho-l-serine; PG: Phosphoglycerol; PI: phospho-myo-inositol headgroups. SM and CH are sphingomyelin and cholesterol, respectively.

**Table 2:**
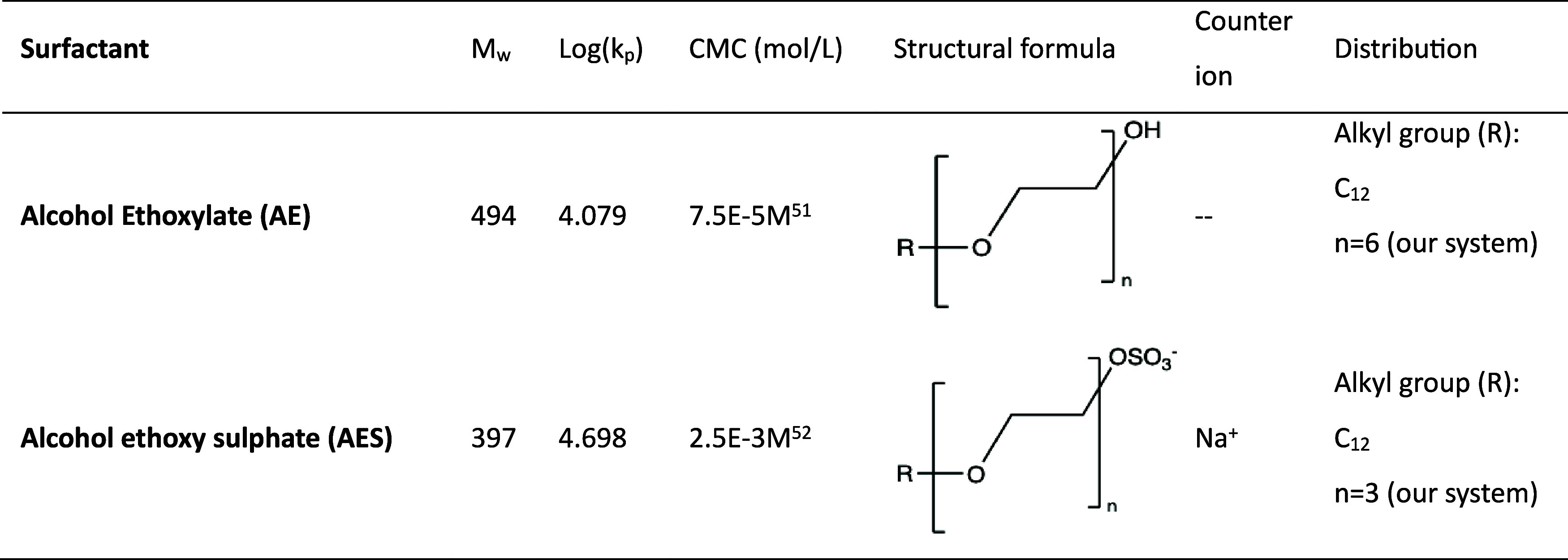
Surfactant Properties Used in this Study^51,52^

The liposomes were constructed with a diameter of 20 nm (Figure 1c), consistent with experimentally observed sizes for similar systems.^17^ During the seeding process using Packmol, water molecules were incorporated to ensure the proper solvation of the lipid bilayer and surfactants. To balance excess charge, counterions were added to the system based on the number and location of charged phospholipid headgroups, considering their distribution between the inner and outer leaflets of the liposome.

### Parameterization

The Packmol^53^ software package was used for initial seeding. The phospholipid molecules were used from the MARTINI v2.0 library.^54^ However, for the ethoxy (EO) groups, MARTINI v3.0 ^49^ was used to obtain the relevant coordinates and topology files alongside other MARTINI v2.0 components for correct parametrization. For CG parametrization of surfactants, CGparam^55^ was utilized that allowed the generation of CG mappings compatible with MARTINI v2.0. The CGparam tool, using SMILES input, produced the necessary structure and topology files, mapping all-atom representations to analogous larger beads (Figure 2a,b). Both AE and AES contain a C12 alkyl chain. However, their CG bead assignments differ in the polar region. In AE, the alkyl chain is connected to a neutral ethoxylate headgroup. In AES, it connects to an ethoxylate chain, ending in a charged sulfate group. This difference alters the effective polarity and interfacial characteristics of the adjacent fragments. Marrink et al. describe such bead assignments driven by partitioning free energies (the “top-down” approach).^54,56^ In a following study from the same group, they also indicated that extending the model to MARTINI 2 resulted in slightly less accurate agreement for certain free energies of transfer compared to the MARTINI 3 model.^57^ Specifically, for PS–PEO block copolymer liposomes, when mapping atomistic (AE) to coarse-grained (CG) representations, we also observed that aggregation characteristics did not perfectly align with the experimental behavior. To address this issue, we modified the AE topology by incorporating corresponding EO beads from Martini 3.0, which were absent from the standard Martini 2.0 ^57^. All the simulation analysis was computed using MDAnalysis,^58^ and simulations were visualized with VMD.^59^

**Figure 2:**
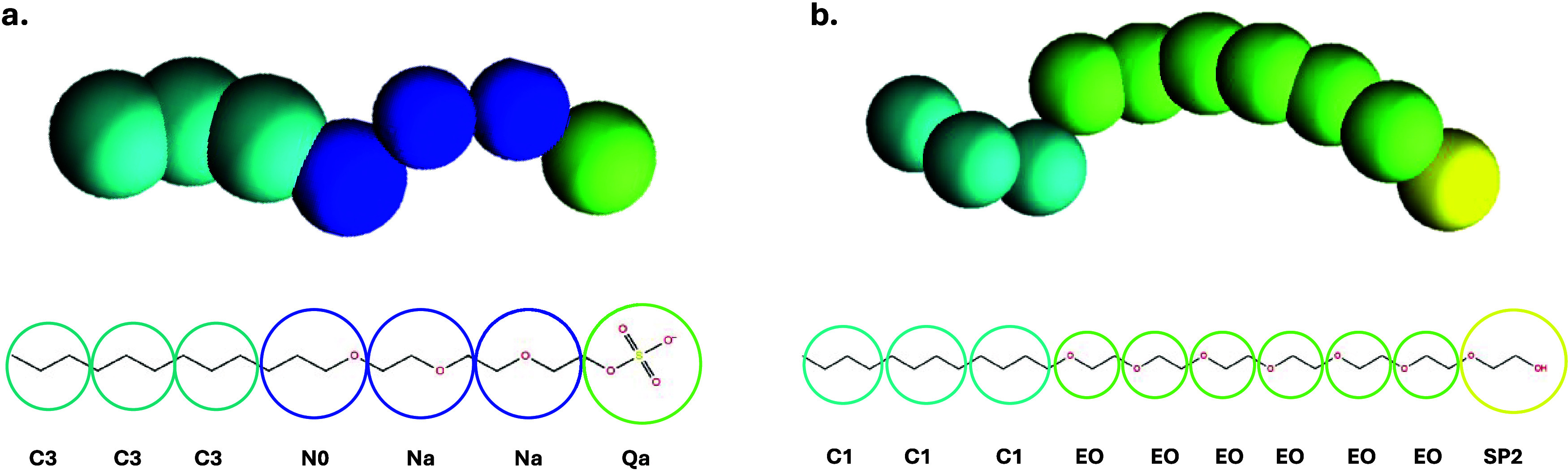
(a) AES all-atom vs coarse-grain parametrized beads, Qa bead as headgroup; (b) AE all-atom vs coarse-grain parametrized beads SP2 bead as headgroup.

### Minimization and Equilibration

A series of equilibration steps were conducted following the initial seeding of molecules to ensure proper relaxation and stability of the system. Energy minimization was done sequentially in an isothermal–isobaric ensemble (NPT) for 0.5 ns and 298 K, a 3 ns canonical (NVT) ensemble with restraints on the liposome to ensure the liposome does not break, 3 ns NPT with restraints on the liposome, and 3 ns NPT without restraints to relax the system. Finally, a 3 μs production run was at 298 K in the NPT ensemble. Energy minimization used the steepest descent algorithm by employing a Verlet cutoff scheme with a tolerance of 0.005.^60^ For electrostatic interactions, we used the Particle Mesh Ewald (PME) method^61^ with a cutoff of 1.1 nm and a relative dielectric constant of 15. Equilibration in NPT was performed using Berendsen barostat^62^ with a coupling constant of 12.0 ps and a semi-isotropic pressure coupling, and a 5 fs time step. Temperature coupling was achieved using the velocity-rescale thermostat^63^ with a coupling constant of 1.0 ps. NVT ensemble used a time step of 3 fs, maintaining temperature control with the v-rescale thermostat. For both NPT and NVT simulations, we employed the reaction-field method for electrostatics with a cutoff of 1.1 nm and a relative dielectric constant of 15. The Verlet cutoff scheme was used for neighbor searching, with a tolerance of 0.005. All NPT simulations were carried out at 298 K.

## Results and Discussion

### Surfactant Self-Assembly

The coarse-grained bilayer liposome, composed of DPPC, DOPE, DOPS, and cholesterol, is initially populated with two types of surfactants, AES or AE. Surfactants are introduced in two configurations: within the outer leaflet (in-liposome seeding, IS) and outside the liposome (outer seeding, OS) (Table 3). The initial seeding is performed using Packmol. Each system is equilibrated and simulated for at least 2 μs using GROMACS. Snapshots taken at different timesteps illustrate the overall transport of surfactants and the structural evolution of the liposome during this timescale. Figure 3a (for AES) and Figure 3b (for AE) presents four snapshots depicting the self-assembly process of surfactants, specifically for AES 6.52% OS and AE 6.89% OS. Since this process occurs only in outer-seeded liposomes, these snapshots focus on OS systems.

**Figure 3:**
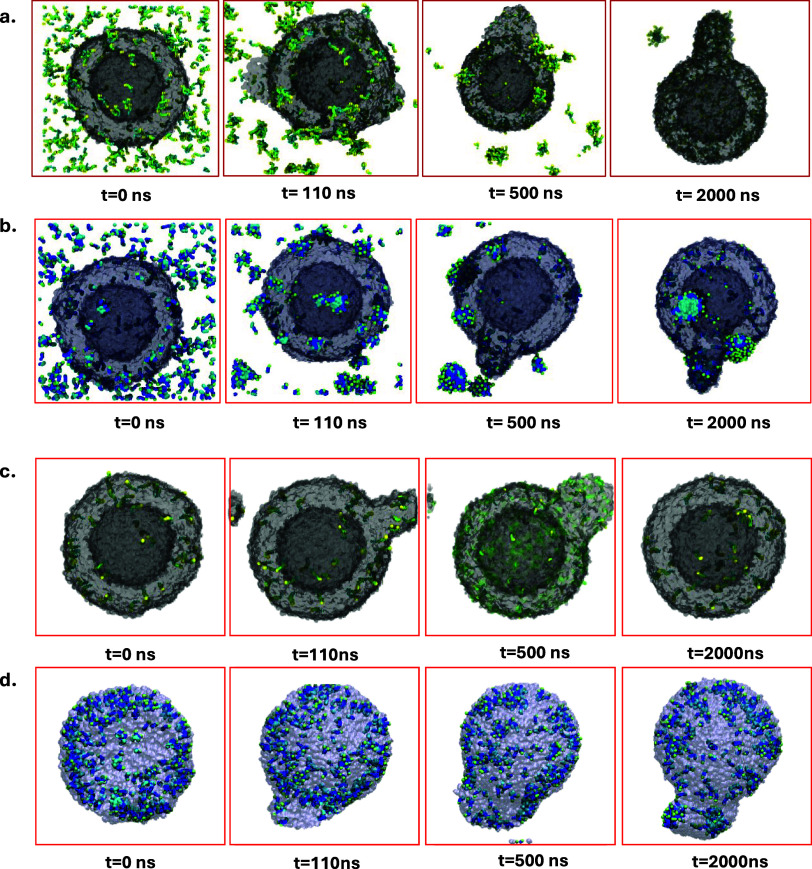
Time-dependent evolution of surfactant–liposome interaction (all front view). (a) AE 6.89% OS, (b) AES 6.52% OS, (c) AE 1% IS, (d) AES 5% IS. Snapshots of key stages observed during our simulation.

**Table 3:** Surfactant Compositions in the Systems and Their Nomenclature in This Work

surfactant	initial seedling location	% surfactant	abbreviation
Alcohol ethoxy sulfate (AES)	Inside liposome, outer leaflet	1	AES 1% IS
		5	AES 5% IS
		10	AES 10% IS
	Outside liposome	1.30	AES 1.3% OS
		6.52	AES 6.52% OS
		13.03	AES 13.03% OS
Alcohol Ethoxylate (AE)	Inside liposome, Outer leaflet	1	AE 1% IS
		5	AE 5% IS
		10	AE 10% IS
	Outside liposome	1.30	AE 1.3% OS
		6.89	AE 6.89% OS
		13.79	AE 13.79% OS
Control	0	No surfactant	

Consistent bulge formation is observed in the outer leaflet of spherical liposomes across all investigated systems as surfactant–liposome interactions progress (Figure 3a–d). The partitioning of surfactant molecules into liposomes results in outward protrusions in the lipid bilayer, as shown in Figure 3a,3b. The bulging phenomenon corresponds to the second stage of Lichtenberg et al.’s three-stage membrane solubilization model, indicating that at the molecular level, a composition-induced disintegration of one of the bilayers forms a long string of thread-like mixed monolayer.^35^

In the system containing 6.89% AE seeded outside the liposome, bulge formation is detected after approximately 100 ns, coinciding with the initial interactions between surfactants and the outer monolayer. Free surfactant monomers, rather than micelles, are identified as the primary species binding to the liposome membrane (Figure 3a,3b), analogous to stage 3 of Lichtenberg et al.’s membrane disruption model.^35^ A correlation is proposed between the degree of bulging and the curvophilic nature of the surfactant as local perturbations in lipid packing increase curvature and generate outward protrusions.

The continued growth of these bulges can be attributed to several complementary mechanisms: (1) surfactant accumulation induces a mass imbalance between the monolayers, amplifying local curvature; or (2) lipid reorganization in response to surfactant presence that potentially leads to phase separation or domain formation that further expands the bulges. Either one of these mechanisms or a combination thereof can be responsible for the observed bulges.

For the outer-seeded systems with over 6% surfactant concentration, transient micelle formation can be noticed consistently. A VMD-rendered visualization detailing this micellization and subsequent membrane-absorption sequence is provided in Supporting Figure S1. To quantify this self-assembly process, we tracked the number of surfactant micelles over time (Supporting Figure S2). The micelle count over time reveals that free surfactant monomers aggregate into multiple smaller micelles early in the simulation. This micellization effect is more pronounced and occurs more rapidly for the nonionic AE compared to the anionic AES. As the simulation progresses, these clusters coalesce and progressively partition into the liposome bilayer, eventually reducing to a single micelle before complete absorption. This effect is more pronounced for AE than AES, as the micelles form sooner. Regardless of initial morphology, all surfactant molecules were absorbed within 2.5 μs (Figure 3a,b), demonstrating that free monomer partitioning serves as the dominant mechanism driving surfactant incorporation into liposomes. This observation supports Hansen et al.’s sequential staged partitioning process, in which surfactant molecules first absorb to the outer monolayer before translocating to the inner leaflet.^16^ A positive correlation can also be noted between surfactant concentration and absorption time, as higher concentrations require longer simulation time for absorption of all surfactants. Because this bulging phenomenon is observed in both the preseeded (IS) and naturally partitioning (OS) systems, it confirms that the curvature is a physical thermodynamic response to the area expansion of the outer leaflet upon surfactant accumulation, rather than an artifact of the initial system setup.

In addition to visual snapshots, the position of surfactants was quantified by measuring their average distances from the dynamic center of mass (COM) of the liposome. The average surfactant distance is compared to the average outer leaflet distance, as shown in Figure 4a–c (for AE) and Figure 4d–f (for AES). These profiles demonstrate that, initially, the average surfactant distance from the COM falls within the 150 Å–175 Å range. Across all systems, a progressive decrease in surfactant distance is observed over time. This trend aligns with the snapshots, indicating that as surfactants self-assemble and partition into the liposome, their distances from the COM decrease. This reduction in the average distance between surfactants and the COM of the liposome serves as an indicator of surfactant partitioning into the liposome when positioned in the solution outside of the liposome. These findings reveal the molecular mechanisms driving early surfactant–liposome interactions. The observed bulge formation represents the system’s response to competing curvature preferences between curvophilic surfactants and curvophobic phospholipids. The preferential partitioning of monomers rather than micelles into the membrane highlights the thermodynamic favorability of monomer insertion, even in solutions containing micellar structures and agree with surfactant monomer adsorption model for micelle and monomers known widely in surfactant chemistry community.^64^

**Figure 4:**
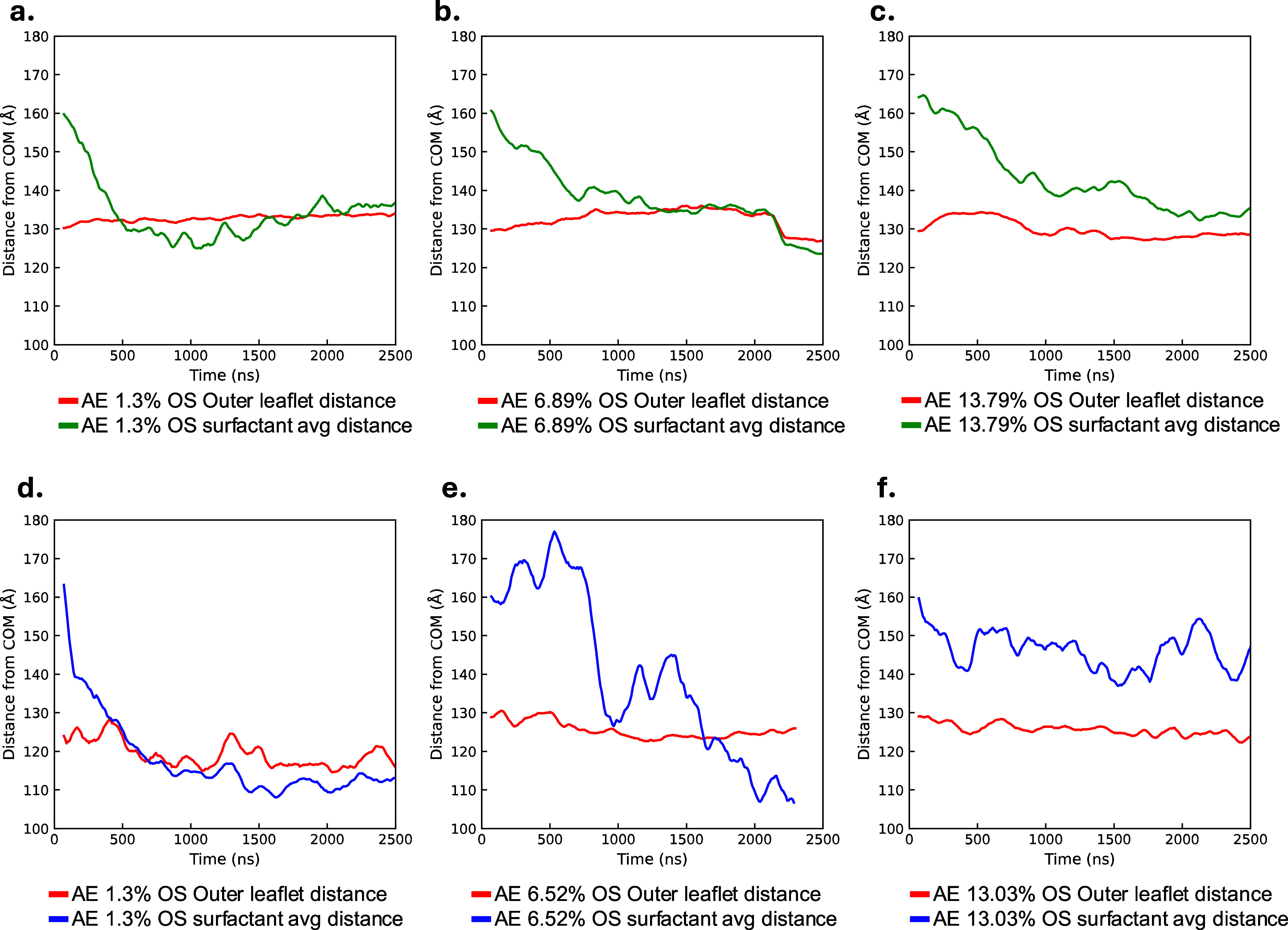
Surfactant transport in the outer-seeded (OS) systems with AE (a−c) and AES (d−f), The plot shows the distance of the molecules, where the average distance of the surfactant is compared to the average distance of the outer leaflet. The comparison shows how the average distance of the surfactants reduces with time progression.

### Inner Liposome Disruption

When individually visualizing the trajectory of the inner and outer leaflets, it was evident that a bulge formed on the outer leaflet of the liposome for all systems (Figure 3a–d). At the same time, the inner leaflets had also undergone disruption as time progressed (Figure 5a,b). Figure 5c demonstrates the inner leaflet’s perforation while the outer layer remains unruptured. Interestingly, the bulge formed on the outer leaflet was always seen on the opposite side compared to the perforation (Figure 5d). In our simulations, AE, with its relatively higher ethoxylation (*n*_EO_ = 6) and nonionic headgroup, caused more frequent and substantial disruptions inside the liposome compared to AES, which possesses a shorter ethoxylate chain (*n*_EO_ = 3) and an anionic headgroup.

**Figure 5:**
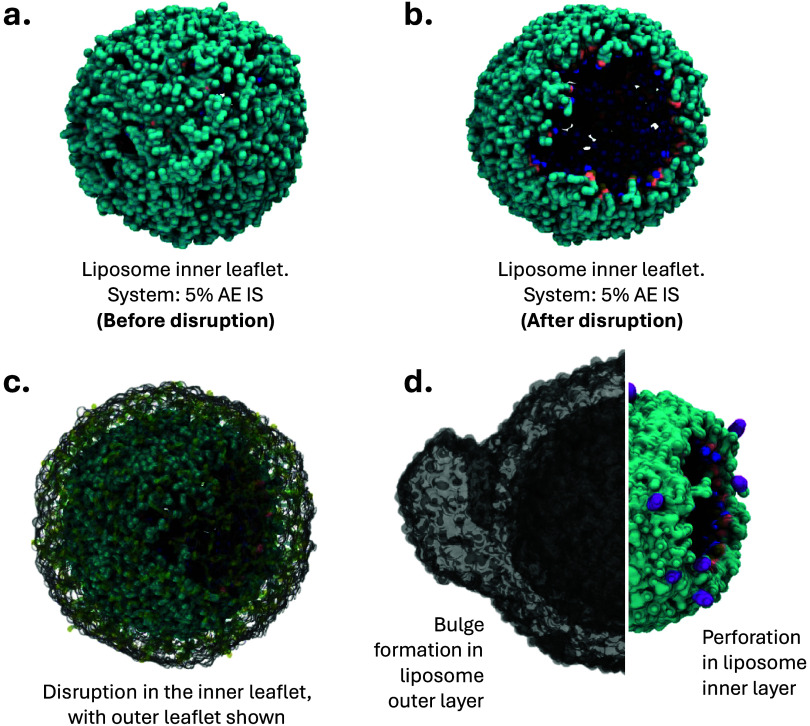
Visual inspection of liposome inner leaflet disruption, illustrating only the inner leaflet of the liposome. The cyan beads represent the hydrophobic tail, and the dark blue beads represent the hydrophilic head of the phospholipids. (a) before disruption (b) after disruption (front view of the liposome). (c) Inner and outer leaflets after disruption in the inner leaflet. (d) Clipped image of a bulge formation on the outer leaflet and inner leaflet disruption of the same liposome (side view of the liposome).

One established method to assess the degree of membrane disruption is by measuring the number of water molecules present inside the inner leaflet of the liposome over the simulation trajectory,^65^ as the number of water molecules present inside the liposome is positively correlated with the liposome inner radius. In this investigation, the positions of water molecules were measured relative to the liposome’s dynamic center of mass (COM).

1 $$N_{w}^{\mathrm{out}\to \mathrm{in}}\left(t\right)=\sum_{k=1}^{N_{w}^{\mathrm{out}}\left(0\right)}\left(\left|r_{k}\left(t\right)-R_{\mathrm{COM}}\left(t\right)\right|<R_{\mathrm{in}}\left(t\right)\right)$$

Here, *N*_w_^out→in^(*t*) is the number of water molecules initially located outside the liposome that are found within the inner leaflet boundary at time t. *N*_w_^out^(0) is the total number of water molecules initially outside the liposome; *r*_*k*_(*t*) is the position of water molecule *k* at time *t*, *R*_COM_ *(t)* is the instantaneous center of mass of the liposome; *R*_in_(t) is the average radius of the inner leaflet at time *t*; Using this relation, water molecules that were initially seeded outside the liposome were tracked over time, with their distances from the COM being measured at each time point to quantify the number of molecules that were transported across the membrane barrier. Figure 6 illustrates the total number of molecules transported over time. All systems exhibited a steady increase (positive slope), with 0.1 to 1% of the outer-seeded molecules transporting inside after 2 μs (Figure 6, primary *y*-axis). However, some of the systems show a sudden and steep increase in the total water molecule count. This steep increase can be related to the inner leaflet disruption, as the perforation allowed easier molecular transport. Thus, the steep change in the molecule count denotes the point of leaflet disruption.

**Figure 6:**
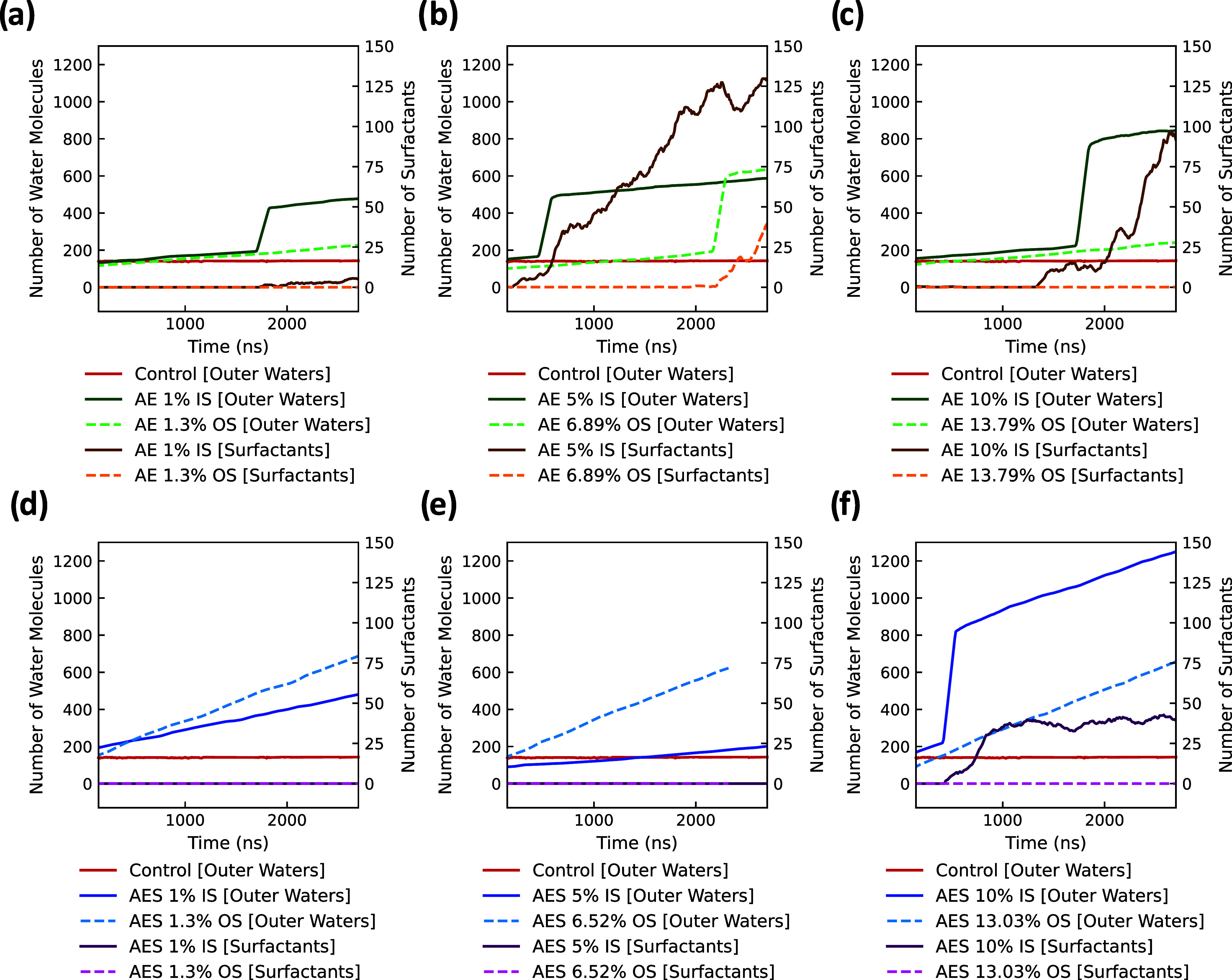
Time-dependent analysis of water and surfactant transport across the liposome bilayer. All systems exhibit a steady increase in water molecule internalization. The top row displays AE systems, with equivalent CMC paired and plotted together (a–c). The bottom row shows AES systems (d–f). The “outer waters” labeled profiles refer to the number of water molecules present outside of the liposome’s inner leaflet radius at each time point. The “surfactant” labeled profile shows the number of surfactant molecules similarly counted. A sharp increase in the water transport profiles indicates sudden disruptions of the leaflet. The secondary axis quantifies the total number of similarly transported surfactant molecules (AE/AES).

Further analyzing the outer water count vs time profile reveals that the systems with AE surfactants consistently demonstrated the inner leaflet disruption for all concentrations (Figure 6a–c). Moreover, the disruptions are from the systems with Inside-liposome seeded (IS) surfactant systems only; the outside-liposome seeded (OS) surfactant systems did not show such a steep increase nor any inner leaflet disruptions (Figure 6a–c, primary *y*-axis). On the contrary, the AES surfactant systems showed inner leaflet disruption at the highest concentration (AE 10% IS) (Figure 6f), while the lower concentration systems and the OS systems did not within the total simulation time of 3 μs (Figure 6d–f).

Additionally, the surfactant’s movements were tracked in a similar manner to count the number of surfactants transported inside the inner leaflet radii. The total surfactant count was plotted on the secondary *y*-axis in Figure 6 to show the correlation between liposome disruption and surfactant count. The surfactant counts reveal that a small number of surfactant molecules (∼20) began to travel inside the leaflet simultaneously, along with the sharp increase in the water transport profile. It is also evident that OS systems did not show such surfactant transport, thus resulting in no inner leaflet disruptions.

This bidirectional diffusion was also observed with water molecules initially seeded inside the liposome (Supporting Information Figure S3), where 3 to 5% of water molecules initially within the inner leaflet were eventually observed outside the structure. This was only observed for surfactant systems and not the control system, confirming that water diffusion occurs through the increased permeability of the lipid bilayer due to the presence of surfactants. It should also be noted that the no-surfactant liposome remained structurally stable over the simulation timescale and showed no abrupt increase in water uptake into the vesicle, in contrast to the disrupted AE- and AES-containing systems.

It was demonstrated by Groot et al.^66^ that the incorporation of surfactants such as C_9_E_8_ and C_12_E_6_ into lipid bilayers leads to the formation of transient and permanent pores, by which membrane integrity is compromised. They identified the hydrophobic chain length as one of the factors in transient pore formation, with more significant disruption being caused by C12-tailed surfactants than by shorter C9 chains. This specific observation aligns with recent experimental literature. Hu et al. systematically investigated alkyl ethoxylate surfactants and demonstrated that the degree of ethoxylation (EO) dictates membrane-lytic activity and subsequent eye irritation.^67^ They concluded that surfactants with intermediate ethoxylation (such as *n*_EO_ = 6) rapidly fuse into lipid membranes and cause severe pore formation, whereas shorter chains (*n*_EO_ ≤ 4) cause only mild structural changes. The behavior of our simulated systems reflects this experimentally established phenomenon: the intermediate-ethoxylated AE (*n*_EO_ = 6) exhibited a high disruption rate, whereas the shorter-ethoxylated AES (*n*_EO_ = 3) showed minimal inner leaflet disruption, except at specific high-concentration inner-seeded conditions. Due to bulge formation, an imbalance between the surface area between the outer and the inner leaflet can cause a transient curvature induced stress in the inner leaflet, that could contribute to the pore formation when the surfactant partition to the leaflet.^67,68^ The absence of disruption in outer-seeded (OS) systems could be attributed to the self-assembly events of the surfactants, which could have delayed or prevented their partitioning into the inner leaflet. It is important to note that membrane perforation is a stochastic process, and the 3 μs simulation timescale employed here is a limiting factor. While this time frame is sufficient to capture the rapid disruption kinetics of the highly active AE systems and the high-concentration AES 10% IS system, it may not capture the equilibrium phase behavior of the slower systems. Given the inherent membrane-active nature of these surfactants, it is highly probable that extended sampling times would reveal similar disruption events in the lower concentration AES systems and the remaining outer-seeded (OS) configurations.

### Membrane Thickness

The thickness of the lipid bilayer influences membrane fluidity, permeability, and the function of embedded proteins. MD simulation enables characterizing membrane thickness during the surfactant–membrane interaction, which is otherwise challenging in in vitro or in vivo scenarios. It has been reported that due to factors such as mechanosensitivity,^69^ lipid composition,^70^ partitioning,^71^ and membrane fluidity,^72^ bilayer membranes undergo thinning processes. In our investigation, the liposome membrane thickness was measured throughout the simulation trajectory by individually tracking each leaflet and calculating the distance between them with respect to the center of mass COM, following established geometric protocols for spherical vesicle characterization.(^74^)­

2 $$d_{m}\left(t\right)=\frac{1}{N_{\mathrm{out}}}\sum_{j=1}^{N_{\mathrm{out}}}\left[{\mathrm{r⃗}}_{\mathrm{out},j}\left(t\right)-R_{\mathrm{COM}}\left(t\right)\right]-\frac{1}{N_{\mathrm{in}}}\sum_{i=1}^{N_{\mathrm{in}}}\left[{\mathrm{r⃗}}_{\mathrm{in},i}\left(t\right)-R_{\mathrm{COM}}\left(t\right)\right]$$

Here, *d*_m_(*t*) is the distance of lipid headgroup beads at time *t*. *N*_in_ and Nout are the total number of atoms in the inner and outer lipid leaflets, respectively. *r*_n,*i*_(*t*) and *r*_out,*j*_(*t*) represent the position vectors of the *i*th atom in the inner leaflet and the *j*th atom in the outer leaflet at time *t*.

The evolution of the membrane thickness over time is presented in Figure 7. This figure demonstrates that the mean membrane thickness decreases across all surfactant-containing systems throughout the simulation trajectory. Each subplot compares the inside-liposome seeding (IS) and outside-liposome seeding (OS) configurations for each system. The control system (without surfactants) maintains a relatively stable membrane thickness.

**Figure 7:**
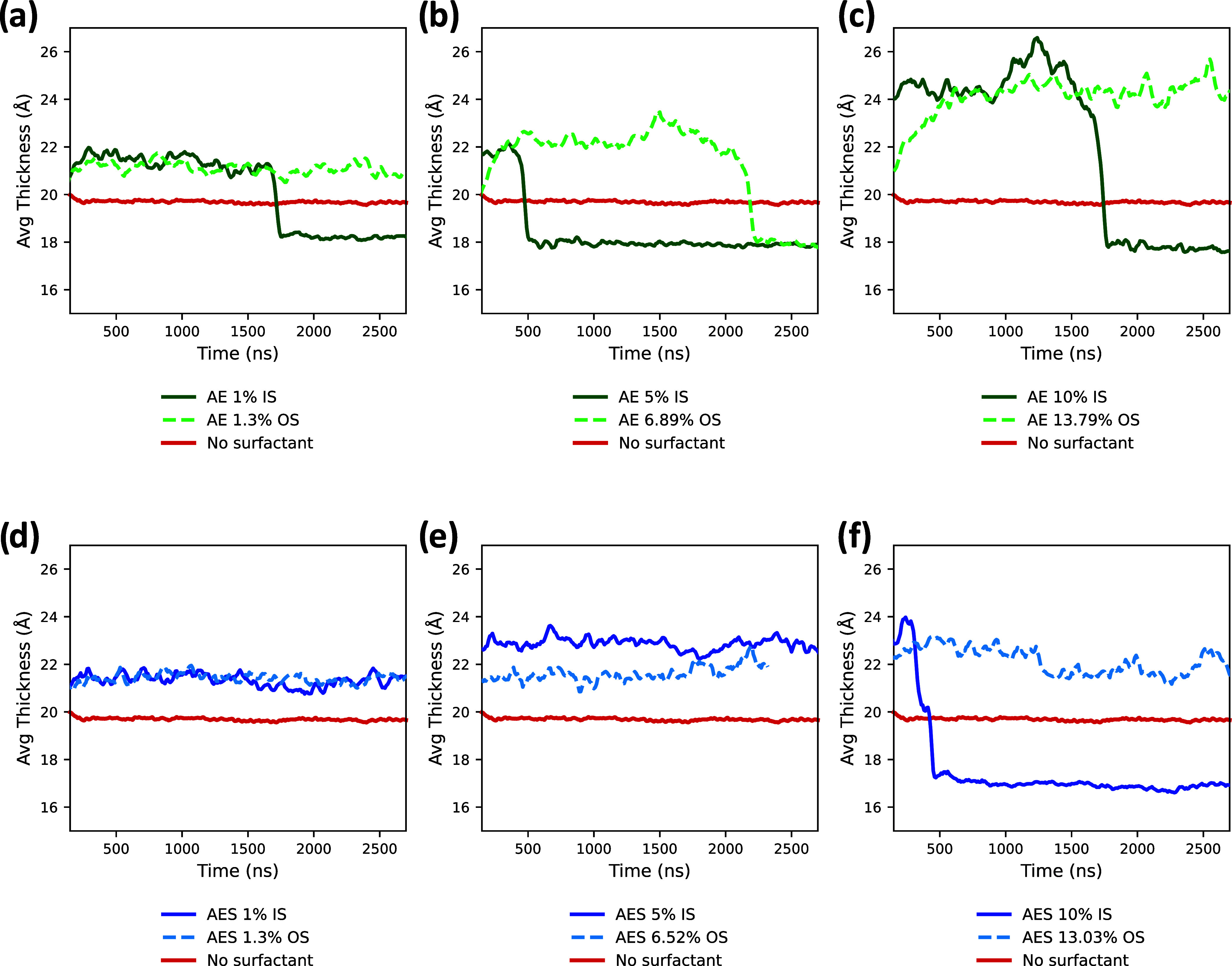
Average membrane thickness across different surfactant-lipid systems over time. Subplots compare inside (IS) and outside (OS) seeding configurations against a stable control baseline. (a–c) AE systems exhibit pronounced thinning corresponding to increased liposome disruption, pore formation, and molecular transport. Conversely, (d–f) AES systems show high structural stability and minimal thickness variations, particularly at lower concentrations (1%–10% IS) and specific outer concentrations (1.3% and 6.52% OS), where inner leaflet disruption is resisted.

The variations in membrane thickness correlate with pore formation in the inner leaflet. In the stable control system and the predisruption surfactant systems, the average membrane thickness is approximately 20 Å (with the most frequent thickness values remaining within the 15–23 Å range, as quantified by the mode distribution in Supporting Figure S5). Against this baseline, systems experiencing inner leaflet disruption show sharp thickness decreases of 2–8 Å (Figure 7a,b). This represents a significant 10% to 40% reduction in local bilayer thickness, highlighting the severe structural compromise of the membrane. At the specific time points when inner leaflet disruption occurs, a sharp decrease in thickness is observed (Figure 7a–c), similar to patterns seen during molecular transport processes in Figure 7. Several systems did not exhibit inner leaflet disruption, including AES at low concentrations (1%, 5%, 10% IS), AES at specific outer seeding concentrations 1.3% OS, and 6.52% OS (Figure 7d–f), and AE at both low and high outer seeding concentrations (1% and 13.79% OS) (Figure 7a,c).

In our observations, surfactants self-assemble and gradually partition into the liposome throughout the simulation. In a recent study, Maeki et al. demonstrated though a time-resolved small-angle X-ray scattering (SASX) that POPC forms bilayer thickness around 42Å.^73^ They replicated the scenario using CG MD simulation to achieve thicknesses about ∼33–34 Å. Our analyzed thickness agrees with their claim, albeit slightly smaller values due to other factors such as having multiple lipid components and having slightly different ensemble conditions. Hu et al. described that surfactant-induced pore formation causes membrane thickness reduction.^67^ The surfactant insertion into the liposome necessitates expansion of the outer leaflet area, contributing to membrane thinning. Additionally, bulge formation in the outer leaflet further contributes to the overall thinning process and explains the variations in mean thickness observed in systems such as AE 10% IS (Figure 7b).

### Surfactant Adsorption Kinetics

To quantify the adsorption of surfactants onto the liposome, the time-dependent surface coverage, Γ­(*t*) (expressed in molecules/nm^2^), was calculated for both the inner and outer leaflets. At each trajectory frame, the dynamic headgroup boundary surfaces (*R*_in_ and *R*_out_) were determined using the fifth and 95th percentiles of the lipid headgroup radial distances from the liposome center of mass.

A surfactant molecule was classified as “bound” to the outer leaflet if its center of mass was located within a cutoff distance of 8.0Å from *R*_out_ and above the membrane midplane. Conversely, it was considered bound to the inner leaflet if it was within 8.0 Å of *R*_in_ and below the midplane. The instantaneous surface coverage for the targeted leaflet was then calculated as

3 $$\Gamma \left(t\right)=\frac{N_{\mathrm{bound}}\left(t\right)}{4\pi R{\left(t\right)}^{2}}\times 100$$

where *N*_bound_(*t*) is the number of adsorbed surfactant molecules at time *t*, *R*(*t*) is the instantaneous radius of the respective leaflet in Å, and the factor of 100 converts the area from Å^2^ to nm^2^. This time-resolved metric enables the tracking of adsorption kinetics until thermodynamic equilibrium (plateau) is reached.

Analysis of the surface coverage (Γ) reveals differences in the binding affinities of the two surfactants (Figure 8). Across all matched concentrations, the nonionic AE systems exhibited significantly higher maximum surface coverage than their anionic AES counterparts. For example, at the highest concentration, AE 13.79% OS reached a dense coverage of ∼0.23 nm^–2^ (Figure 8a–c), whereas AES 13.03% OS was saturated at only ∼0.11 nm^–2^ (Figure 8d–f).

**Figure 8:**
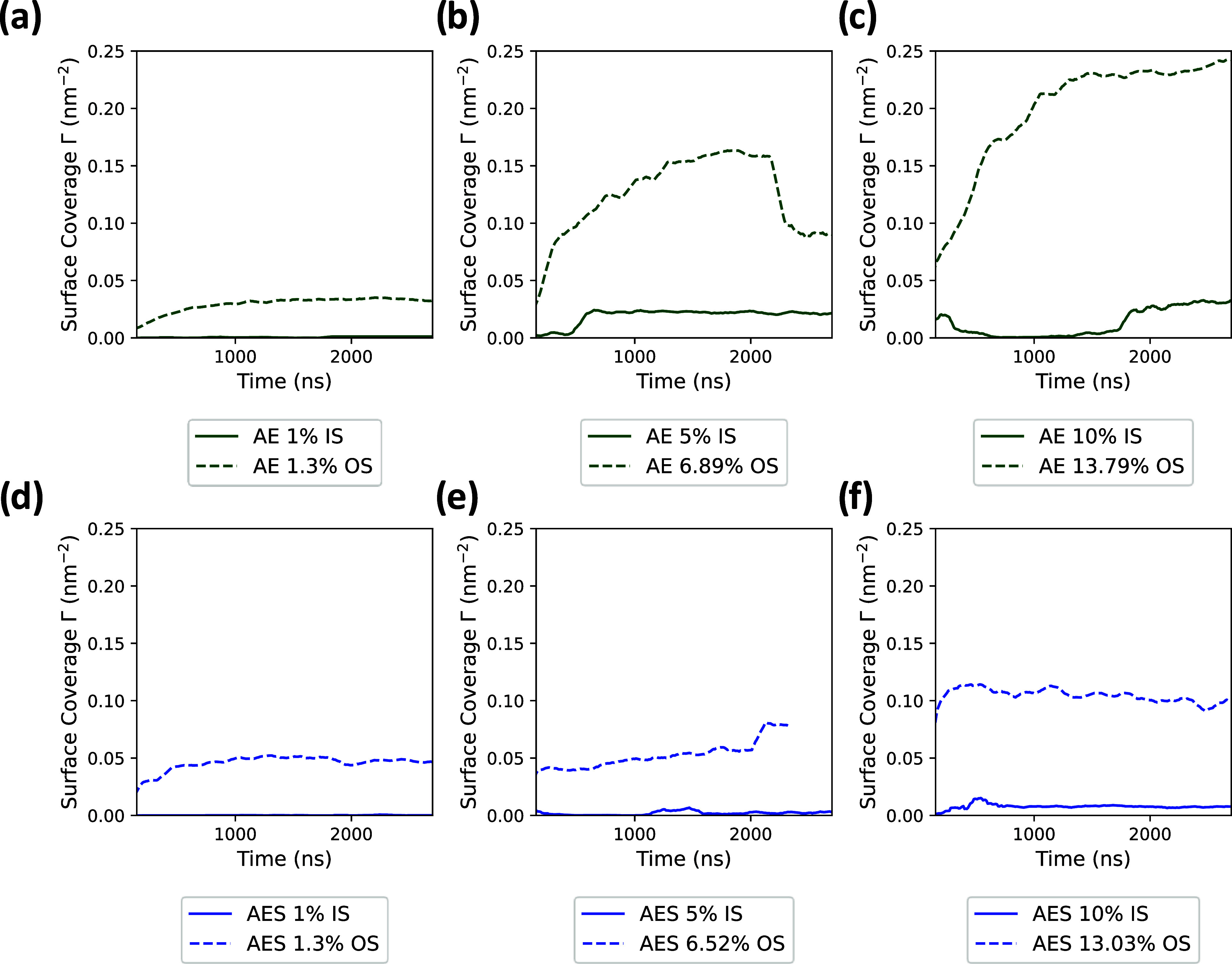
Time-dependent surfactant surface coverage, Γ­(*t*), on the liposome membrane. (a–c) AE systems and panels. (d–f) AES systems at increasing surfactant concentrations for in-liposome/outer-leaflet seeded (IS) and outside-liposome seeded (OS) configurations. Surface coverage was calculated as the number of bound surfactant molecules normalized by the instantaneous leaflet surface area.

This phenomenon is governed by the electrostatics of the surfactant headgroups. AE features an uncharged ethoxylate headgroup, allowing the molecules to tightly pack laterally at the lipid–water interface with minimal penalty. In contrast, AES contains a negatively charged sulfate group. As AES molecules accumulate on the membrane, they exert strong lateral electrostatic repulsion against one another (and against anionic DOPS lipids in the membrane). This repulsive penalty actively resists dense packing, forcing the AES adsorption to plateau at a much lower surface coverage. It should be noted that the current simulations were performed in a charge-neutralized environment without additional background ions. The presence of physiological salt concentrations (e.g., ∼150 mM NaCl) would likely induce Debye screening, potentially reducing this repulsive penalty and altering both adsorption kinetics and critical micelle concentrations. Therefore, incorporating physiological salt composition remains an important variable for future in silico ocular membrane models.

As expected from standard adsorption isotherm behavior, increasing the nominal concentration of AE (from 1% to 10%) resulted in a proportional increase in surface coverage, demonstrating that the membrane has a high carrying capacity for the uncharged surfactant before reaching complete saturation.

Furthermore, outside-seeded (OS) systems consistently achieved an order of magnitude higher surface coverage than inside-seeded (IS) systems. This discrepancy is attributed to two factors: (1) compartment reservoir size: The outer bulk water volume is vastly larger than the internal lumen, providing a much larger absolute number of surfactant monomers available for partitioning onto the membrane, and (2) membrane curvature: The outer leaflet possesses a convex curvature, which spreads the lipid headgroups slightly apart and creates hydrophobic defects that facilitate surfactant insertion. Conversely, the concave curvature of the inner leaflet forces the lipid headgroups into tighter packing, introducing a steric barrier that physically hinders surfactant adsorption.

### Lateral Diffusion

Aside from vertical translocation within the lipid bilayer, lateral movement (diffusion) of lipids serves as a primary indicator of membrane fluidity and structural integrity.^40^ Because surfactants disrupt the tight packing of curvophobic phospholipids, we hypothesized that their incorporation would increase overall membrane fluidity. To quantify this, we calculated the lateral diffusion coefficients. Lateral diffusion on the curved surface of a spherical liposome is a two-dimensional process. Therefore, the lateral diffusion coefficient (*D*_lat_) was calculated using the 2D Einstein relation:^75^

4 $$\left\langle r^{2}\right\rangle =4\mathrm{Dt}$$

Here, ⟨*r* ^2^⟩ represents Mean Square Displacement (MSD), *D* is the diffusion coefficient derived from the Einstein relation, and *t* denotes time. This approach follows established methodologies for calculating lateral diffusion on spherical vesicles.^76^ These simulations were performed in the NPT ensemble to observe structural changes, so these diffusion values should be interpreted as qualitative comparative metrics rather than absolute NVE transport coefficients. The results (Figure 9) demonstrate that the baseline lipid diffusion in the pure liposome (no surfactant) is highly restricted (∼0.6 × 10^–7^ cm^2^/s). Upon the introduction of surfactants, lipid mobility increases significantly across all systems. For the nonionic AE systems, lipid and surfactant lateral diffusion coefficients remain relatively stable and closely coupled (ranging between 2.0 and 3.5 × 10^–7^ cm^2^/s) regardless of concentration or seeding method. This indicates that AE effectively partitions into the bilayer, thoroughly integrating with the lipids and fluidizing the membrane uniformly. In contrast, the anionic AES systems exhibit highly variable and drastically elevated diffusion coefficients, particularly in the outer-seeded (OS) configurations (reaching up to 15 × 10^–7^ cm^2^/s). This disparity highlights the difference between diffusion inside the tight membrane environment versus diffusion in the bulk aqueous phase. Surfactants in pure water typically diffuse orders of magnitude faster than those constrained within a lipid bilayer. The exceptionally high apparent diffusion in AES OS systems suggests that, due to the electrostatic repulsion of the sulfate headgroups, many AES molecules remain in the bulk water phase or form transient, highly mobile micelles that do not fully partition into the stable lipid bilayer within the simulated time frame. This dynamic micelle partitioning, where aggregates form, collide with the membrane, and potentially extract lipids into mixed micelles, thus creates a much higher average mobility compared to the deeply integrated AE molecules.

**Figure 9:**
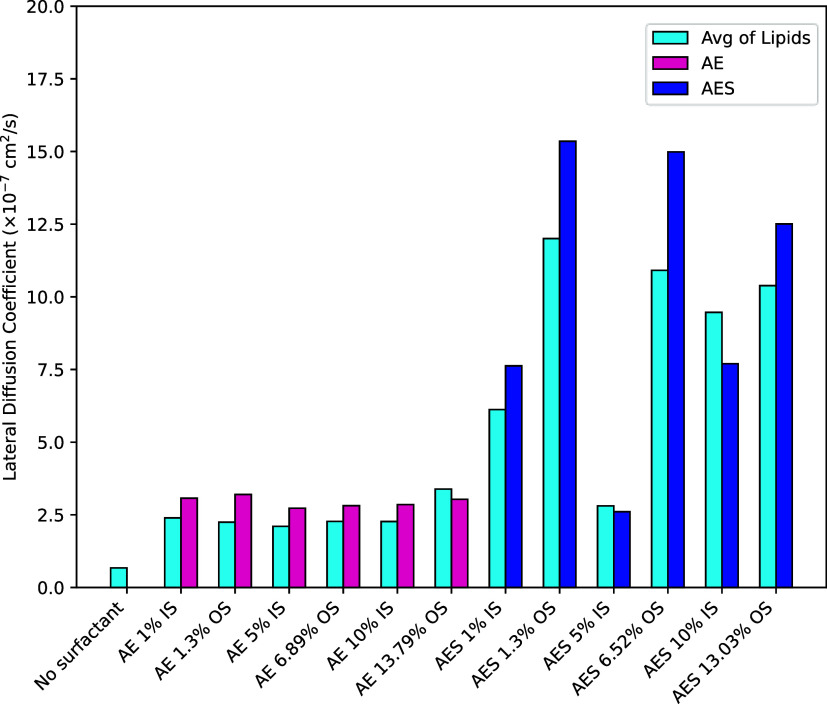
Lateral diffusion coefficients across systems with varying surfactant types and concentrations. The comparison shows enhanced lateral lipid mobility in bilayers upon surfactant introduction.

While these diffusion trends provide valuable mechanistic insights, it is important to note the boundaries of the current computational model. The coarse-grained models for AE and AES were constructed using the standard building-block approach of the Martini force field, which relies on matching thermodynamic partitioning data. While this approach is widely adopted and has been validated for similar polyoxyethylene and sulfated amphiphiles, we note that explicit validation against experimental monolayer surface tensions or all-atom adsorption free energies, such as the rigorous benchmarking performed for the SPICA/SDK force fields (Shinoda et al., 2010; 2011),^77,78^ was not performed in this study. Consequently, while the models successfully capture the qualitative interfacial behaviors and relative steric differences between AE and AES, the absolute values of the lateral diffusion coefficients should be interpreted as semiquantitative. Future studies incorporating explicit surface tension validation (e.g., compared to experimental data by Rincón-Romero et al.,^79^ and Varadaraj et al.,^80^) would further refine the transferability of these specific surfactant parameters.

## Conclusion

This study presents a comprehensive coarse-grained molecular dynamics investigation into how nonionic (AE) and ionic (AES) surfactants interact with liposomes that mimic corneal epithelial cell membranes. By simulating large-scale, biologically representative liposome systems under various surfactant concentrations and seeding configurations, the structural and dynamic consequences of surfactant partitioning into the bilayer membranes were characterized. Our results demonstrate that both surfactants (AE and AES) induce curvature-driven bulge formation on the liposome outer leaflet. However, the nonionic head-grouped and higher ethoxylated AE molecules impart more consistent liposome perforation, resulting in increased water permeation and higher membrane thinning. Both surfactants impart significantly higher (<5×) lateral lipid mobility, resulting in a more fluid lipid lateral diffusion within their respective leaflets. The nonionic and higher ethoxylated AE (*n*_EO_ = 6) molecules showed a higher (<20%) diffusion coefficient than their AES (*n*_EO_ = 3) counterpart systems, indicating a higher lipid mobility across the simulation trajectory.

These findings provide molecular-level insights into how amphiphilic surfactants perturb the epithelial cell membranes. Furthermore, leveraging the CG MD system enables simulation of a 10 to 100 times increase in overall simulation timescale and reduced computational resources due to fewer degrees of freedom calculation and larger timesteps.^28,54,55^ In this study, CG MD combined with Packmol-based initial configuration was employed to eliminate uncertainties in self-assembled bilayer liposome formation. This approach enables efficient simulation in observing the liposome–surfactant interaction dynamics. The interaction observed in these simulations aligns with the experimental reports on surfactant-induced ocular toxicity and membrane solubilization mechanisms.^28,47^ Additionally, this approach offers a simplistic *in silico* framework for evaluating new surfactant candidates when designing surfactant-containing product formulations. While the current study successfully differentiates the aggressive disruption of AE from the milder behavior of AES under outer-seeded conditions, establishing a fully predictive *in silico* screening framework will require future validation.

## Supplementary Material



## Data Availability

Data supporting the findings of this study are available from the corresponding author upon reasonable request.

## Notes

Detailed molecule and ion count for all simulated alcohol ethoxylate (AE) and alcohol ethoxy sulfate (AES) systems; methodologies and time-dependent trajectories for bulk surfactant micelle cluster analysis; inner water molecule transport tracking profiles highlighting bilayer leaflet disruptions; visualization of liposome self-interaction across periodic boundary conditions; and mode membrane thickness distributions over time (PDF)
