# Supplementary material for: Molecular Modeling of Surfactant Interaction on Phospholipid Bilayers Mimicking Corneal Epithelium
Source: J Chem Theory Comput. 2026 Jun 29;22(15):7966–80. doi: 10.1021/acs.jctc.6c00863 (PMC13472081; doi:10.1021/acs.jctc.6c00863)
Supplement: Supplementary file 1 [file ct6c00863_si_001.pdf]

Supplementary information

For

Molecular modelling of surfactant interaction on phospholipid bilayers mimicking corneal epithelium

Isteaque Ahmed[1], Stephen J Fox[2], C Mark Maupin[3], Oreste Todini[4], Dilnoza Amirkulova[5]

[1]Procter and Gamble Digital Accelerator at University of Cincinnati, Cincinnati, OH

[2]Procter and Gamble, Reading RG2 0RX, UK

[3]Pacific Northwest National Laboratory, Richland, WA 99352

[4]Procter and Gamble, Brussels, Belgium

[5]Procter and Gamble, Mason, OH

Contents

Figure S1: Outer seeded surfactant molecule's micelle formation and partitioning into the liposome (AES 1% OS system). 3

Figure S2: Time-dependent micelle analysis for the outside-seeded (OS) surfactant systems. Micelles were identified by clustering bulk surfactant molecules using center-of-mass distances, with only clusters containing at least 5 molecules counted as micelles. 5

Figure S3: Time-dependent analysis of inner water molecules' transport across the liposome bilayer. The top row displays AE systems, with equivalent CMC paired and plotted together. The bottom row shows AES systems. The "inner waters" labeled profiles refer to the number of water molecules present within the liposome's inner leaflet radius at each time point. Sharp increase in the water transport profiles indicate sudden disruptions of the leaflet. 6

Figure S4: liposome self-interaction across the periodic bounding box for system AES 5% (IS) at 200ns. 7

Figure S5: mode of thickness distribution (the most frequently occurring thickness value at each timepoint). AE systems on the top row and AES system on the bottom row. 8

Table summarizing the molecules counts in the systems:

Table S1: Detailed molecule counts of the systems

System	AE	AES	CHOL	DOPE	DOPS	DPPC	Water (10% antifreeze)	Cl^-	Na^+
**AES_1.3_outside**	0	76	155	531	437	2002	378939	4326	5225
**AES_10_inside**	0	586	155	531	437	2002	378939	4326	6245
**AES_13.03_outside**	0	764	155	531	437	2002	378939	4326	6601
**AES_1_inside**	0	58	155	531	437	2002	378939	4326	5189
**AES_5_inside**	0	293	155	531	437	2002	378939	4326	5659
**AES_6.52_outside**	0	382	155	531	437	2002	378939	4326	5837
**AE_1.3_outside**	61	0	155	531	437	2002	378939	4326	5073
**AE_10_inside**	471	0	155	531	437	2002	378939	4326	5073
**AE_13.79_outside**	650	0	155	531	437	2002	378939	4326	5073
**AE_1_inside**	47	0	155	531	437	2002	378939	4326	5073
**AE_5_inside**	235	0	155	531	437	2002	378939	4326	5073
**AE_6.89_outside**	324	0	155	531	437	2002	378939	4326	5073
**Control**	0	0	155	531	437	2002	378939	4326	5073

Surfactant absorption by the vesicle/reduction in micelle count:

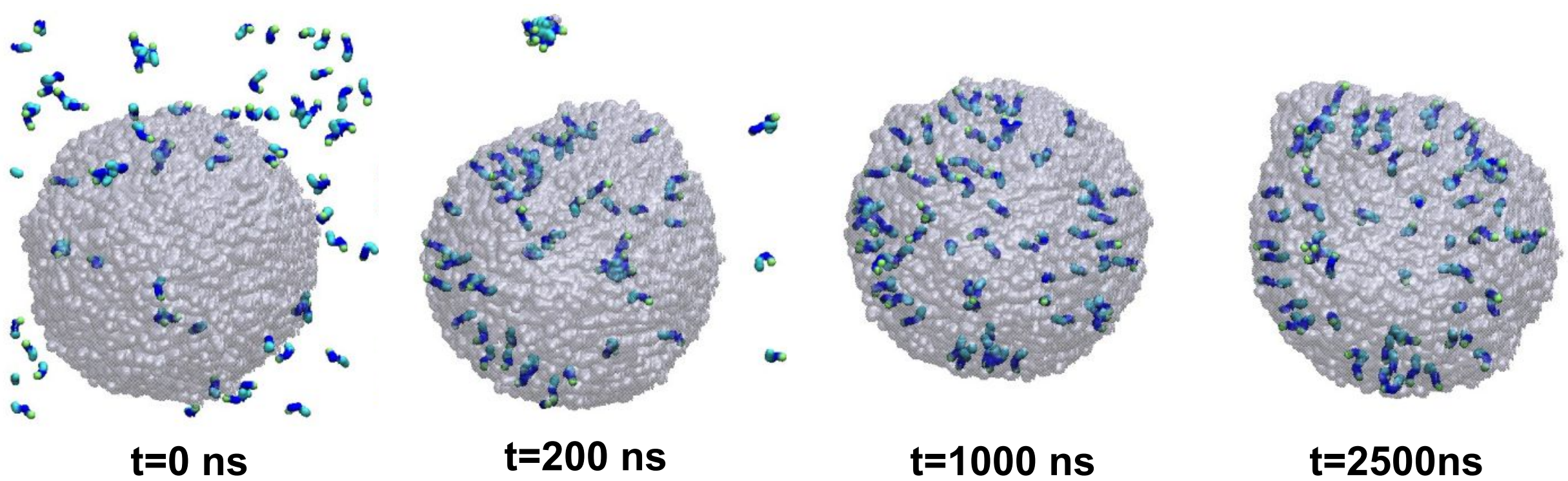

Figure S1: Outer seeded surfactant molecule's micelle formation and partitioning into the liposome (AES 1% OS system).

Micelle counts:

To quantify transient surfactant micellization in the outside-seeded (OS) systems, we performed a frame-by-frame cluster analysis using the center of mass (COM) of each surfactant molecule. At each trajectory frame, the instantaneous liposome center was defined from the inner leaflet lipids, and the

inner and outer membrane boundaries were estimated from the 5th and 95th percentiles of the radial lipid distributions, respectively. Surfactant molecules were classified as bulk surfactants if their COM lay outside the membrane region, i.e., either inside the lumen below the inner boundary or in the external solution beyond the outer boundary, using an additional membrane buffer.

Bulk surfactants were then grouped into clusters based on a COM–COM distance cutoff, and clusters were identified using connected-component analysis of the resulting adjacency matrix. Only clusters containing at least 5 surfactant molecules were counted as micelles, in order to exclude isolated monomers and very small transient aggregates. The micelle-count analysis showed that, in OS systems, surfactants first formed multiple small aggregates in the bulk phase, followed by progressive coalescence and eventual loss of bulk micelles as surfactants were absorbed into the membrane (Figure S3). Thus, the number of micelles generally decreased with time, while the average micelle size transiently increased during coalescence before dropping as the bulk surfactant population was depleted.

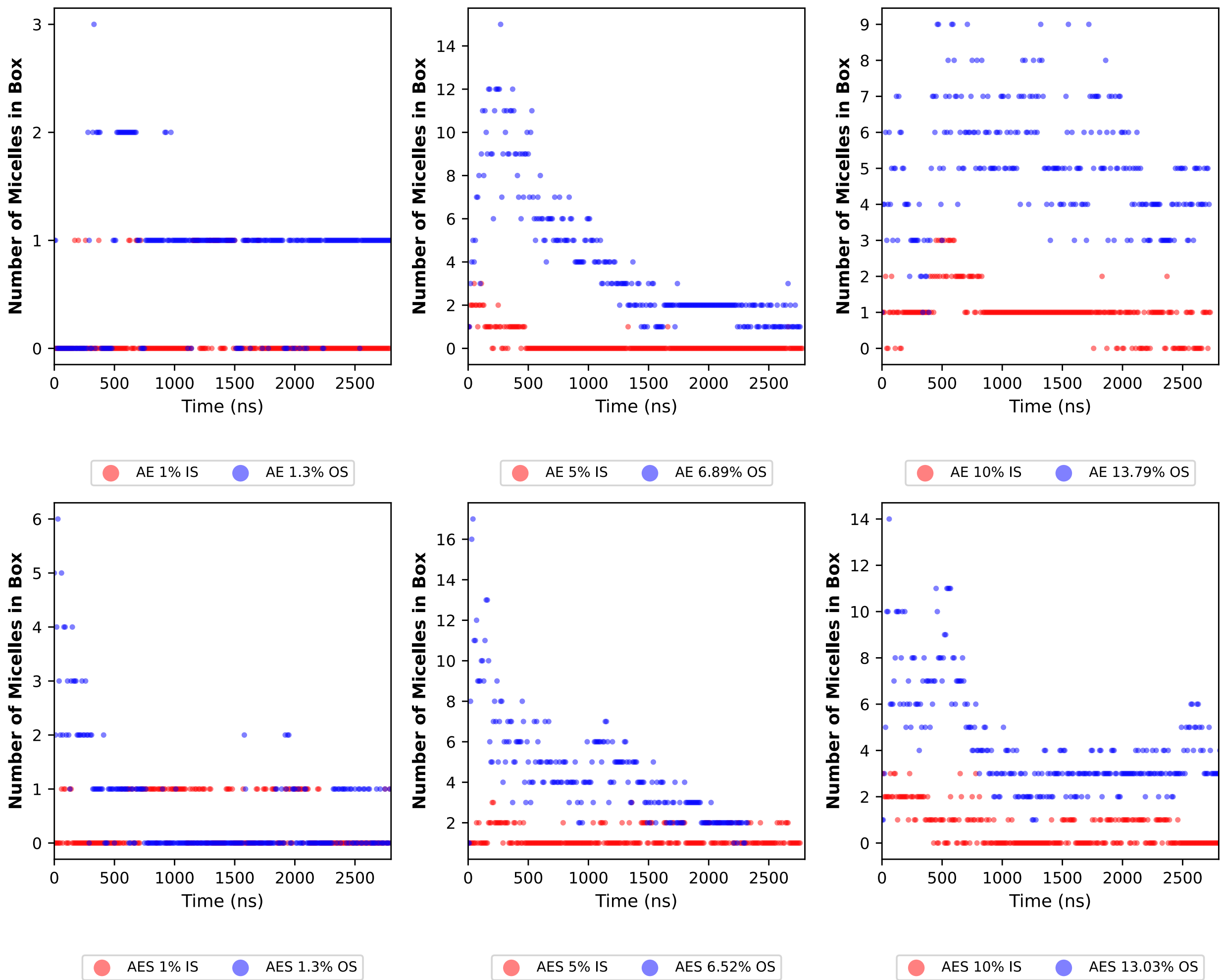

Figure S2: Time-dependent micelle analysis for the outside-seeded (OS) surfactant systems. Micelles were identified by clustering bulk surfactant molecules using center-of-mass distances, with only clusters containing at least 5 molecules counted as micelles.

Tracking water molecules initially seeded inside the inner leaflet radius:

Tracking inner water molecules across the trajectory. The number of water molecules present within the inner leaflet (calculated with reference to the dynamic COM) at specific timepoints were counted. The plot demonstrates the relative relation of molecules between their respective CMC systems. The sudden spikes in molecule count represent the point when the disruption on the inner leaflet occurs.

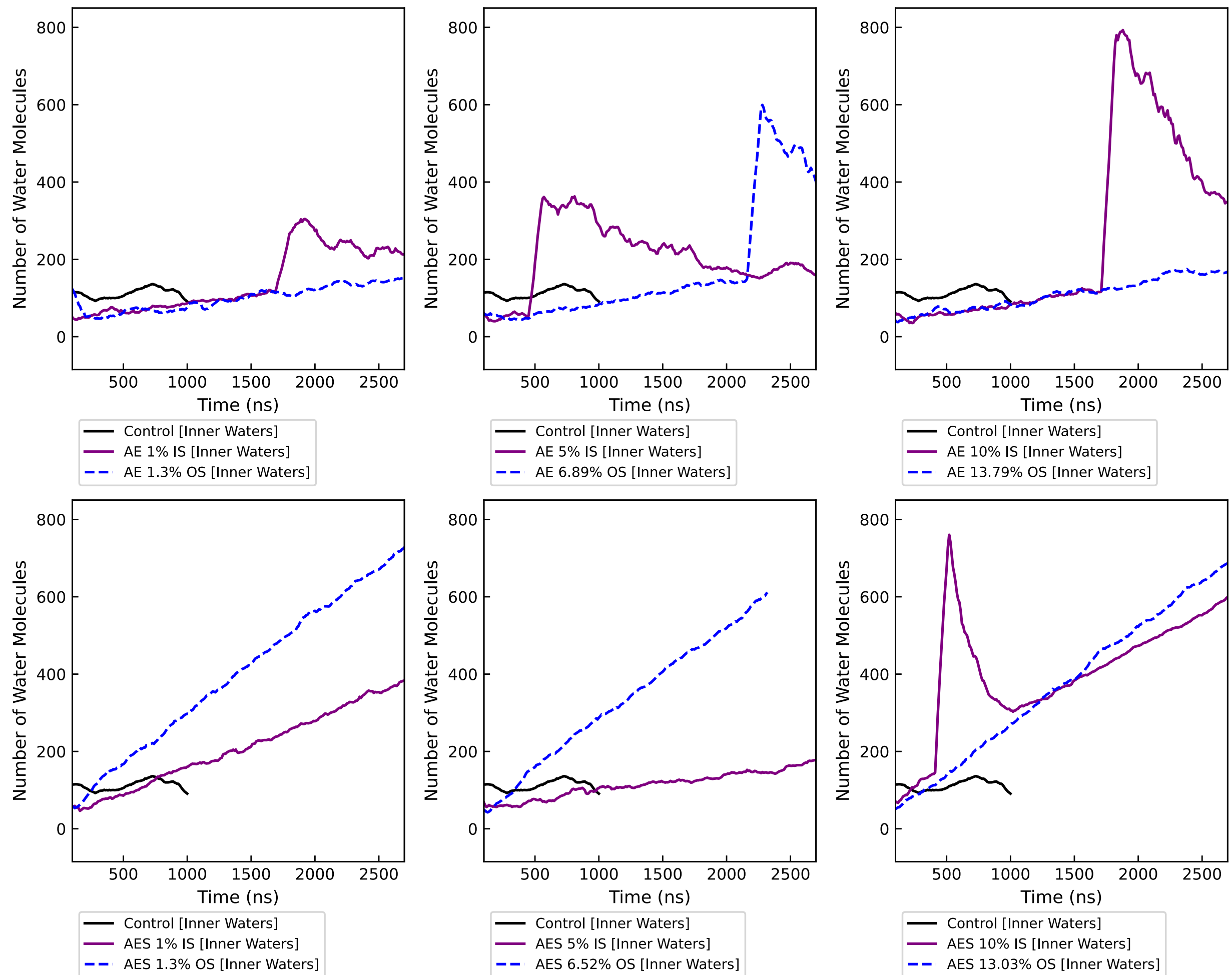

Figure S3: Time-dependent analysis of inner water molecules' transport across the liposome bilayer. The top row displays AE systems, with equivalent CMC paired and plotted together. The bottom row shows AES systems. The "inner waters" labeled profiles refer to the number of water molecules present within the liposome's inner leaflet radius at each time point. Sharp increase in the water transport profiles indicate sudden disruptions of the leaflet.

Self-interaction of liposome:

Following liposome is for system AES 5% (IS) demonstrate liposome self-interaction across the periodic bounding box.

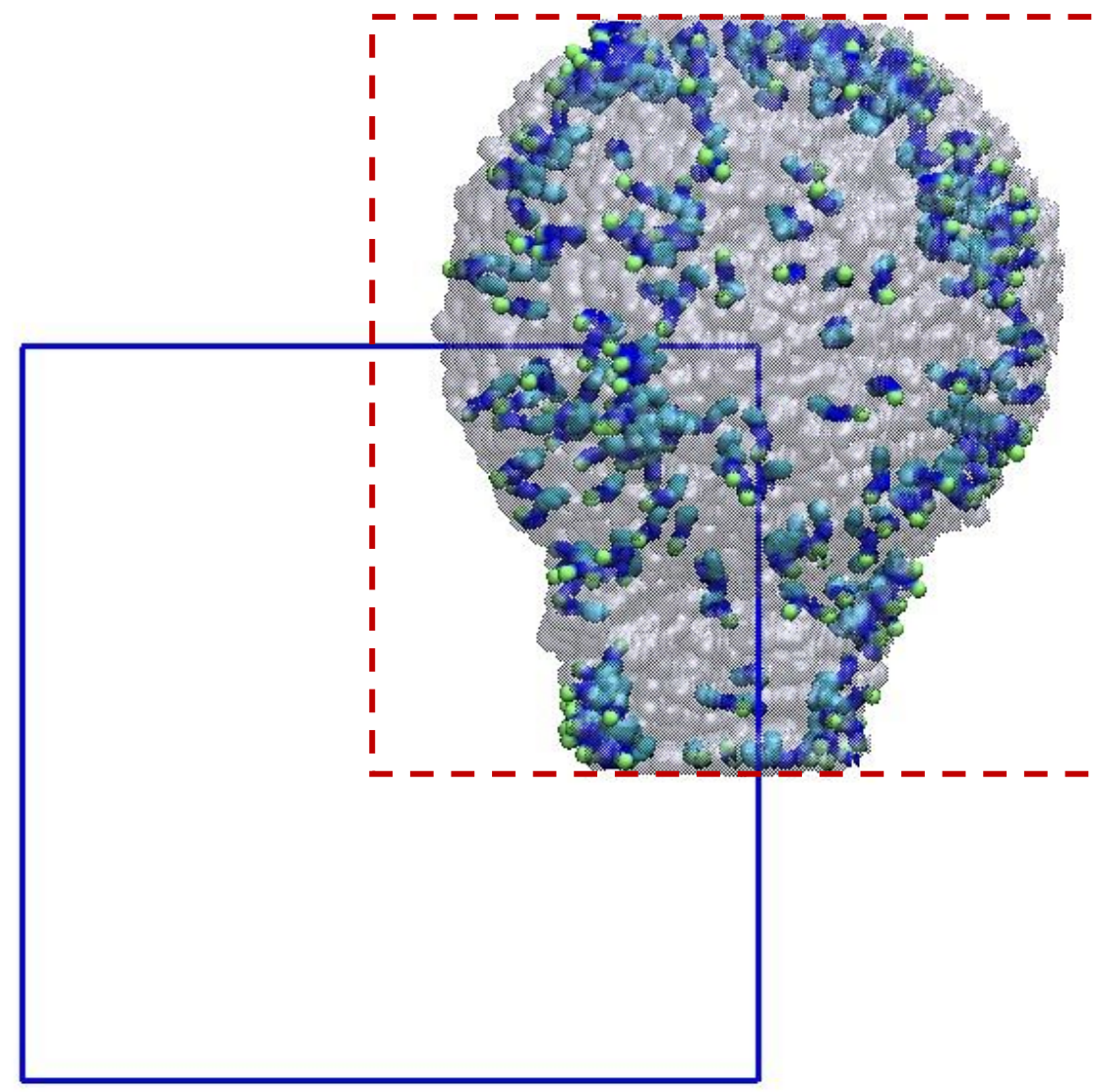

Figure S4: liposome self-interaction across the periodic bounding box for system AES 5% (IS) at 200ns.

Membrane thickness (mode):

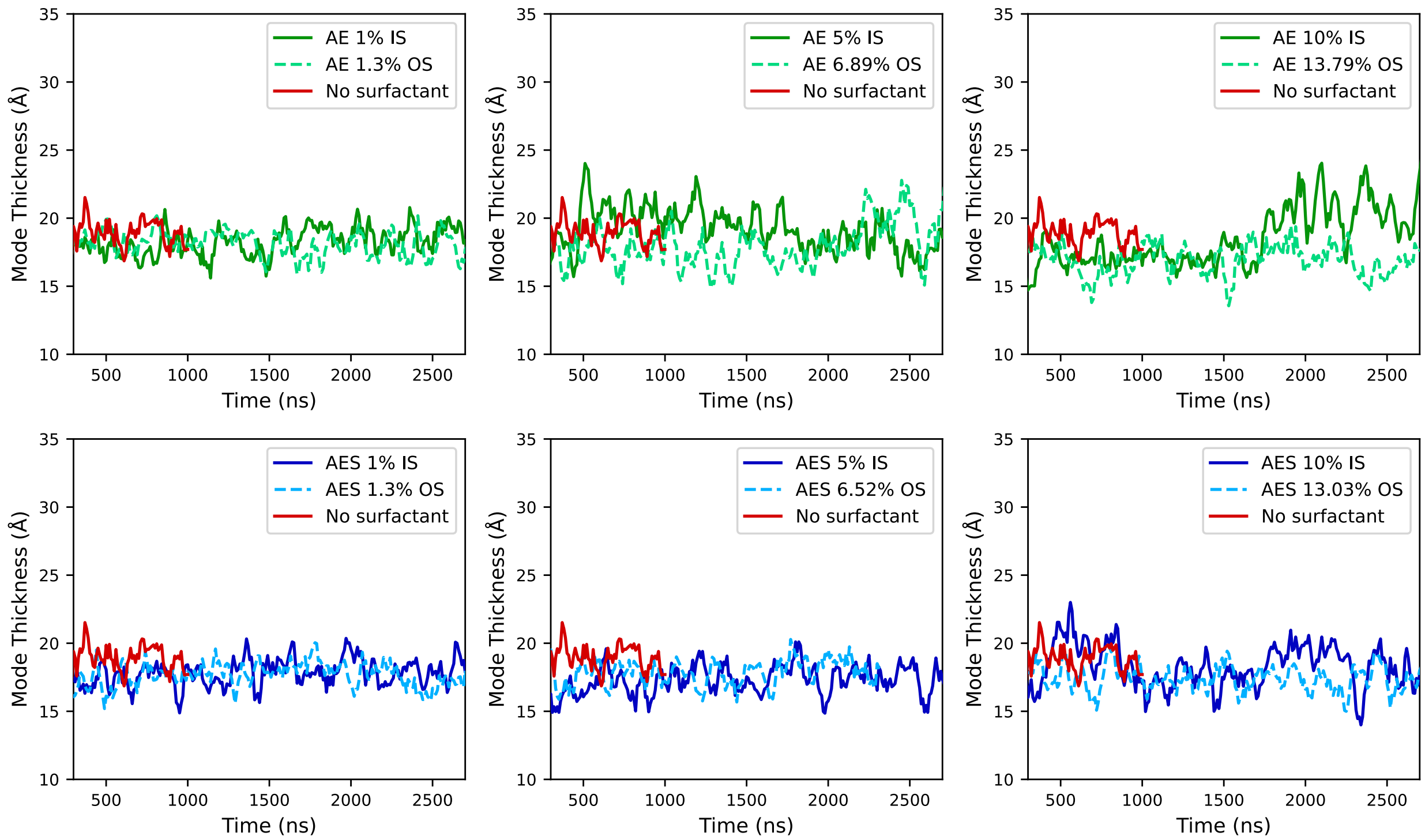

Figure S5: mode of thickness distribution (the most frequently occurring thickness value at each timepoint). AE systems on the top row and AES system on the bottom row.
